# Abnormal Regional Spontaneous Brain Activity and Its Indirect Effect on Spasm Ratings in Patients With Hemifacial Spasm

**DOI:** 10.3389/fnins.2020.601088

**Published:** 2020-12-09

**Authors:** Fei-Fei Luo, Hui Xu, Ming Zhang, Yuan Wang

**Affiliations:** ^1^Department of Medical Imaging, The First Affiliated Hospital of Xi’an Jiaotong University, Xi’an, China; ^2^Institute of Biomedical Engineering, School of Life Sciences and Technology, Xi’an Jiaotong University, Xi’an, China

**Keywords:** resting-state fMRI, hemifacial spasm, fractional amplitude of low-frequency fluctuation, regional homogeneity, degree centrality, mediation analysis

## Abstract

**Purpose:**

Three classical methods of resting-state functional magnetic resonance imaging (rs-fMRI) were employed to explore the local functional abnormalities and their effect on spasm ratings in hemifacial spasm (HFS) patients.

**Methods:**

Thirty HFS patients and 30 matched healthy controls (HCs) were recruited. Rs-fMRI data, neurovascular compression (NVC) degree and spasm severity were collected in each subject. Fractional amplitude of low-frequency fluctuation (fALFF), regional homogeneity (ReHo), and degree centrality (DC) were calculated in the whole brain voxels. Two sample *t*-tests were performed to investigate group differences of fALFF, ReHo, and DC. Correlation analysis was performed to assess the relationships between the regional brain abnormalities and clinical variables in HFS.

**Results:**

Compared with HCs, HFS patients exhibited increased fALFF in the left precuneus and right posterior cingulate cortex (PCC), together with increased ReHo in the bilateral PCC and bilateral precuneus. Decreased ReHo was observed in the right middle occipital gyrus (MOG), right superior occipital gyrus (SOG), right cuneus, and right angular gyrus (AG) in HFS patients. Moreover, ReHo in the right PCC were positively correlated with NVC degree and spasm severity in HFS patients, respectively. Mediation analysis revealed that increased ReHo in the right PCC regulated the neurovascular compression degree, and further resulted in increased spasm ratings.

**Conclusion:**

Our study revealed regional brain dysfunctions from different perspectives and an indirect effect of ReHo in right PCC on spasm ratings predominantly through the alteration of NVC.

## Introduction

Hemifacial spasm (HFS), a rare neuromuscular movement disorder, is characterized by unilateral, involuntary, and intermittent facial muscle contractions innervated by the ipsilateral facial nerve (Jannetta, 1998; Wang and Jankovic, 1998). It initially affects the orbicularis oculi muscle and then gradually extends to the whole hemifacial muscles (Wang and Jankovic, 1998; Au et al., 2004; Rosenstengel et al., 2012). Although HFS is not a life-threatening disease, the patients with HFS often suffer from visual and verbal disabilities, social embarrassment, depression, and anxiety (Tan et al., 2004, 2005; Rosenstengel et al., 2012). It is generally considered that the neurovascular compression (NVC) at root exit zone of facial nerve induces HFS (Moller, 1991). However, surgical failure and spasm recurrence make it necessary to conduct more research from different aspects to deeply understand the pathophysiological mechanisms of HFS, which can improve the diagnosis and treatment for this disorder (Pleger, 2016).

Previous studies have demonstrated gray and white matter abnormalities and glucose hypermetabolism in the brain of HFS patients using structural MRI (Bao et al., 2015; Tu et al., 2016; Guo et al., 2019; Xu et al., 2019) and positron emission tomography (PET) (Shimizu et al., 2012). However, only two studies have investigated brain functional alterations in HFS patients through resting-state fMRI (rs-fMRI) until now, which is of advantage because subjects do not need to participate in complicated tasks under clinical conditions (Biswal et al., 1995; Fox and Raichle, 2007). One of the rs-fMRI studies revealed brain network reorganization and amygdala functional abnormalities in HFS patients (Xu et al., 2019), and another study applied regional homogeneity (ReHo) analysis to reveal abnormal ReHo in extensive brain regions of HFS patients (Tu et al., 2015), including the left middle frontal gyrus (MFG), left medial cingulate cortex (MCC), left lingual gyrus, right superior temporal gyrus (STG), right precuneus, left precentral gyrus, anterior cingulate cortex (ACC), right brainstem, and right cerebellum. Nonetheless, there was no study exploring regional brain activity alterations in HFS patients with the combination of fractional amplitude of low-frequency fluctuation (fALFF) (Zou et al., 2008), ReHo, and degree centrality (DC) (Buckner et al., 2009).

ALFF characterizes the spontaneous neural activity intensity at a voxel (Zang et al., 2007; Zuo et al., 2010). Compared with ALFF sensitive to physiological noise, fALFF has higher sensitivity and specificity for detecting spontaneous brain activities (Zou et al., 2008). ReHo reflects the local coherence of spontaneous neural activity and the importance of a voxel in the nearest neighbors (Zang et al., 2004). DC reflects the node characteristic of large scale brain network architecture, and reveals the importance of a voxel among the whole brain voxels (Buckner et al., 2009). Therefore, fALFF, ReHo, and DC progressively reflect the regional brain activities from different perspectives (Lv et al., 2019), and the integration of these metrics could identify local brain abnormalities more comprehensively and accurately. As an example, brain regions exhibiting group differences by ReHo between ADHD patients and healthy controls were not identical to those detected by ALFF, but rather complementary (An et al., 2013).

The previous studies, as mentioned above, suggested functional abnormalities of local brain in HFS patients, especially the cingulate cortex. Therefore, we hypothesized that local brain functions may alter more or less in HFS patients, especially in the cingulate cortex. Then, we applied fALFF, ReHo, and DC to investigate functional alterations from different perspectives in HFS patients. Furthermore, considering surgical failure and spasm recurrence, we also investigated the correlations between these abnormalities with neurovascular compression degree and spasm severity to explore whether dysfunctions in some brain regions were a cause of HFS.

## Materials and Methods

### Participants

This study was approved by the Ethics Committee and in accordance with the Declaration of Helsinki. Written informed consent was obtained from each participant before this study. Thirty HFS patients and 30 age- and sex-matched healthy volunteers were recruited in this study. They were all right handed. The inclusion criteria for HFS were as follows: typical hemifacial muscle spasms with involuntary and intermittent onset, and without additional neurological or sensory deficits. The exclusion criteria included secondary HFS caused by tumors and cysts; alcohol or drug abuse; psychiatric or neurologic disorder history; and MRI contraindications caused by metal implant or claustrophobia. All of the healthy controls had no history of psychiatric or neurologic disorders, alcohol or drug abuse.

### Clinical Variables

Before imaging data acquisition, the disease duration and facial muscles spasm severity assessed by the Cohen spasm scale were obtained for HFS patients from grade 0 to grade 4 according to the following criteria: 0-none; 1-increased blinking caused by external stimuli; 2-mild, noticeable fluttering, not incapacitating; 3-moderate, very noticeable spasm, mildly incapacitating; 4-severely incapacitating (unable to drive, read, etc.) (Cohen et al., 1986). Furthermore, the NVC degree was evaluated by using cranial nerve MRI sequences: 0-no vessel contact; 1-mild contact without compression of the nerve; 2-moderate compression of the facial nerve without deviation of its natural course; 3-severe deviation of the facial nerve (Mauricio and Kaufmann, 2008).

### MRI Data Acquisition

All MRI data were acquired using a GE HDxt 3.0-T MRI scanner (GE Medical Systems, Inc., Milwaukee, WI, United States). Foam paddings and earplugs were applied to minimize head movement and scanner noise. During scanning, all participants were required to keep awake with eyes closed, remaining motionless and relaxed. The three dimensional fast imaging employing steady state acquisition (3D-FIESTA) and time-of-flight magnetic resonance angiography (TOF-MRA) were combined to more accurately visualize local microstructures (Bao et al., 2015; Wang et al., 2016, 2019). The reconstructed 3D-FIESTA images were applied to evaluate NVC degree in the root exit area of facial nerve for each HFS patient (Bao et al., 2015; Wang et al., 2016). Then, high-resolution 3D T1-weighted images were obtained with the following parameters: 136 sagittal slices, thickness/gap = 1.0/0 mm, TR/TE = 10.7/4.9 ms, flip angle = 15°, FOV = 256 × 256 mm^2^, matrix = 256 × 256. Finally, the rs-fMRI data were acquired using gradient echo-planar sequence with the following parameters: TR/TE = 2000/35 ms, flip angle = 90°, 28 slices, thickness/gap = 4.0/0 mm, FOV = 240 × 240 mm^2^, matrix = 64 × 64, 150 contiguous functional volumes. It should be noted that none of the HFS patients had spasm onset during the data acquisition.

### Data Preprocessing

The images were preprocessed using the Data Processing Assistant for Resting-State fMRI (DPARSF V4.4) (Yan and Zang, 2010) based on the Statistical Parametrical Mapping (SPM 12) in MATLAB R2014a (V8.3.0.532). Routinely, the first 10 time points were removed for signal equilibrium and adaptation of participants to the scan. Slice timing correction was performed to correct the acquisition temporal difference between volume slices, and realignment was performed for head motion correction. Structural images were co-registered to the mean function images and then segmented into gray matter, white matter (WM), and cerebrospinal fluid (CSF). Functional images were spatially normalized to the Montreal Neurological Institute (MNI) space with a 3 × 3 × 3 mm^3^ resolution, and smoothed with a 4 mm Full Width at Half Maximum (FWHM) Gaussian kernel with the linear trend removed. Nuisance covariates were regressed out, including WM, CSF signals, and head motion effects using the Friston 24-parameter model. Temporal band-pass filtering (0.01 to 0.08 Hz) was performed except for ALFF and fALFF calculation. It was noted that three HFS patients were excluded due to maximal head motion translation >3.0 mm in any direction or maximal rotation >3.0°. The participants whose head motion parameters mean framewise displacement (FD) (Jenkinson et al., 2002) higher than 2^∗^ group SD (0.1556) above the group mean FD (0.1320) of all the participants (threshold: 0.4432) were also excluded.

### fALFF, ReHo and DC Calculation

All of the following analyses were calculated using DPARSF. ALFF is calculated as the averaged square root of power spectrum within 0.01–0.08 Hz frequency band (Zang et al., 2007). fALFF is calculated as the ratio of ALFF within 0.01–0.08 Hz frequency band to the entire detectable frequency range (Zou et al., 2008). ReHo is calculated as a rank-based Kendall’s coefficient of concordance to measure the local synchronization of the time series of nearest neighboring voxels (usually 27 voxels) (Zang et al., 2004). DC is calculated as the number or weighted sum of significant connections between a voxel’s time course with other voxel’s time course (Buckner et al., 2009; Zuo et al., 2012). In this study, weighted DC was used for its more precise centrality characterization than binary DC (Cole et al., 2010), and the significant connections for calculating DC referred to the correlation coefficient threshold r*_*ij*_* > 0.25(Buckner et al., 2009) eliminate counting voxels with low temporal correlation caused by noise (Buckner et al., 2009). Then, Z-standardization (subtracting the mean fALFF/ReHo/DC value of the entire brain from each voxel and then dividing by the corresponding standard deviation of fALFF/ReHo/DC) of voxel-wise fALFF, ReHo, and DC maps were performed in turn. Finally, Z-standardization maps of ReHo and DC were spatially smoothed with a 4 mm FWHM Gaussian kernel. A whole brain mask was used.

### Statistical Analysis

Demographical and clinical data were analyzed using the Statistical Package for Social Sciences (SPSS 18.0, IBM Inc., Chicago, IL, United States). Group difference in age between HFS patients and healthy controls was tested using two sample *t*-tests, and Chi-square test was used to estimate between-group sex difference. Two sample *t*-tests were performed using the DPARSF to find brain regions showing group differences between patients and controls in fALFF, ReHo, and DC maps. The *T*-maps of fALFF [FWHM = (7.7224 7.8253 6.8346)], ReHo [FWHM = (10.3884 10.8129 10.2793)] and DC [FWHM = (10.3624 10.7074 10.3173)] were thresholded using the Gaussian Random Field theory (GRF) for multiple comparisons with a voxel-level of *p* < 0.01 and then corrected with a cluster-level of *p* < 0.05 in the DPARSF toolbox. The effect size, namely the standardized mean difference, was calculated using the RESTplus V1.24 (Jia et al., 2019). The fALFF/ReHo/DC clusters showing significant group differences were extracted as regions of interest (ROI) masks. Then the Z-standardized fALFF/ReHo/DC values of mean time course in the ROI masks for HFS group were extracted using the DPARSF.

To assess the relationship between fALFF/ReHo/DC and clinical features including neurovascular compression degree and facial spasm severity in HFS group, the above mean Z-standardized fALFF/ReHo/DC values in the ROI masks were inputted to SPSS to calculate the non-parametric Spearman correlation with the clinical variables (Cohen spasm scale and NVC degree). The correlations were considered statistically significant when *p* value was less than 0.05 (i.e., *p* < 0.05). If the correlations were significant, a tentative mediation analysis was performed using the PROCESS macro in SPSS (Hayes, 2013) to explore whether the abnormal fALFF/ReHo/DC in a certain brain region had a direct or indirect effect on spasm ratings through the change of neurovascular compression, where the significance was estimated using a bias-corrected bootstrapping method with 5,000 iterations. 95% confidence interval (CI) not containing zero was used to determine the significant mediating effect. In the mediation analysis, we firstly regarded fALFF/ReHo/DC in a certain brain region as the independent variable, NVC degree as the mediator variable, and Cohen spasm scale as the outcome variable. Then, another corresponding mediation analysis was performed with NVC degree as the independent variable, fALFF/ReHo/DC in this region as the mediator variable, and Cohen spasm scale as the outcome variable.

## Results

### Demographics and Clinical Data

Three patients were excluded for excessive head movement, leaving 27 HFS patients and 30 healthy controls for further analysis. The ratio of HFS patients with right side spasm and left side spasm was 16/11. Demographics and clinical data for these participants were summarized in Table 1. There were no significant differences in age (*p* = 0.84) and sex (*p* = 0.82) between patient and control group. Mean disease duration of HFS patients is 3.45 years. Mean Cohen spasm scale and mean NVC degree in patients were 2.93 and 1.81, respectively.

**TABLE 1:** Demographics and clinical characteristics of all participants.

Characteristic	HFS patients (*n* = 27)	HCs (*n* = 30)	*p*-value
Age (years)	49.77 ± 11.61	50.37 ± 10.94	0.84^*a*^
Sex (female/male)	17/10	18/12	0.82^*b*^
Disease duration (years)	3.45 ± 3.70	NA	NA
Cohen spasm scale	2.93 ± 0.73	NA	NA
NVC degree	1.81 ± 0.79	NA	NA

*HFS, hemifacial spasm; HCs: healthy controls; NVC, neurovascular compression; NA, not applicable. ^*a*^*p* value was obtained using the two-sample *t*-test. ^*b*^*p* value was obtained using the Pearson Chi-square test.*

### Local Functional Alterations in HFS Patients

**FIGURE 1:**
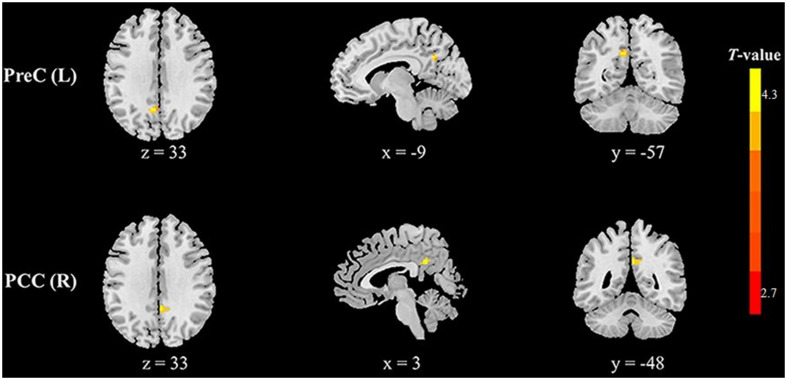
Clusters showing increased fALFF in HFS patients as compared to that in healthy controls (voxel-level *p* < 0.01, cluster-level *p* < 0.05, GRF correction, cluster size > 58 voxels). Left in the figures represents the left side of the brain. PreC, precuneus; PCC, posterior cingulate cortex; L, left; R, right.

**FIGURE 2:**
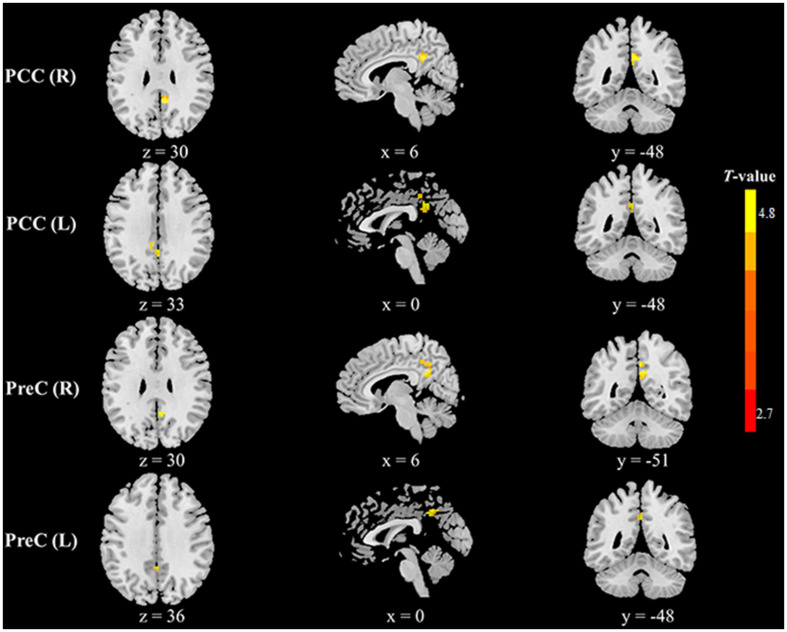
Clusters showing increased ReHo in HFS patients as compared to that in healthy controls (voxel-level *p* < 0.01, cluster-level *p* < 0.05, GRF correction, cluster size > 134 voxels). Left in the figures represents the left side of the brain. PCC, posterior cingulate cortex; PreC, precuneus; L, left; R, right.

**FIGURE 3:**
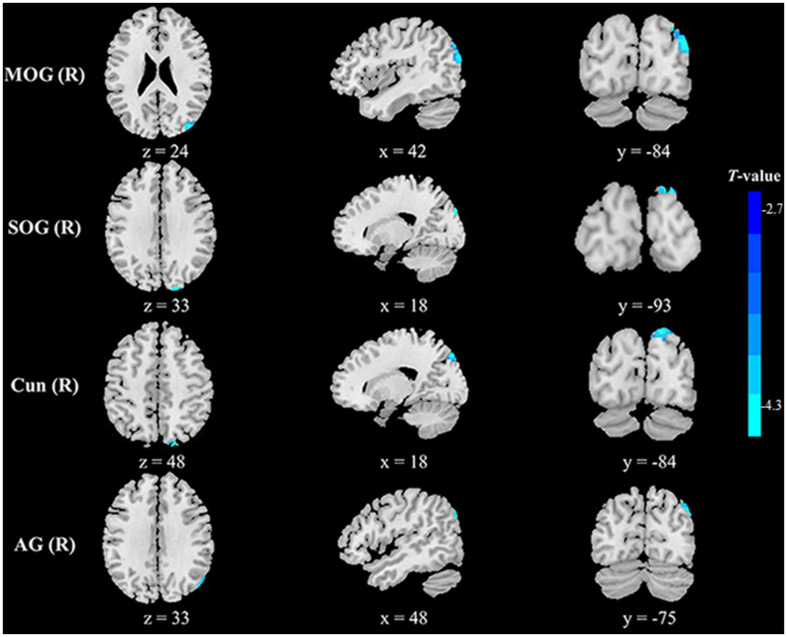
Clusters showing decreased ReHo in HFS patients as compared to that in healthy controls (voxel-level *p* < 0.01, cluster-level *p* < 0.05, GRF correction, cluster size > 134 voxels). Left in the figures represents the left side of the brain. MOG, middle occipital gyrus; SOG, superior occipital gyrus; Cun, cuneus; AG, angular gyrus; L, left; R, right.

Compared with healthy controls, HFS patients exhibited increased fALFF in the left precuneus and right posterior cingulate cortex (PCC) (voxel-level *p* < 0.01, cluster-level *p* < 0.05, GRF correction, cluster size > 58 voxels) (Table 2 and Figure 1). In addition, HFS patients demonstrated increased ReHo in the bilateral PCC and bilateral precuneus (voxel-level *p* < 0.01, cluster-level *p* < 0.05, GRF correction, cluster size > 134 voxels) (Table 2 and Figure 2). Conversely, decreased ReHo was observed in the right middle occipital gyrus (MOG), right superior occipital gyrus (SOG), right cuneus, and right angular gyrus (AG) (voxel-level *p* < 0.01, cluster-level *p* < 0.05, GRF correction, cluster size > 134 voxels) (Table 2 and Figure 3). However, there were no brain regions exhibiting to be significantly different DC between HFS patients and healthy controls (voxel-level *p* < 0.01, cluster-level *p* < 0.05, GRF correction, cluster size > 133 voxels). All original maps of ALFF, fALFF, ReHo, and DC were shared to facilitate subsequent meta-analysis and were available from the corresponding author upon reasonable request.

**TABLE 2:** Brain regions with abnormal fALFF and ReHo in HFS patients compared to HCs.

Regions	Side	Cluster size (voxels)	MNI coordinate (mm)			Peak voxel *T*-value	Effect size
			x	y	z		
**fALFF**							
*HFS* > *HCs*							
PreC	L	17	−9	−57	33	4.392	1.140
PCC	R	16	3	−48	33	3.895	1.011
**ReHo**							
*HFS* > *HCs*							
PCC	R	36	6	−48	30	4.850	1.258
	L	48	0	−48	33	3.864	1.003
PreC	R	42	6	−51	30	4.246	1.102
	L	14	0	−48	36	3.978	1.032
**ReHo**							
*HFS* < *HCs*							
MOG	R	83	42	−84	24	−4.396	−1.141
SOG	R	50	18	−93	33	−3.761	−0.976
Cun	R	35	18	−84	48	−4.314	−1.119
AG	R	31	48	−75	33	−3.578	−0.928

*HFS, hemifacial spasm; HCs: healthy controls; MNI, Montreal Neurological Institute; L, left; R, right; PreC, Precuneus; PCC, posterior cingulate cortex; MOG, Middle Occipital Gyrus; SOG, Superior Occipital Gyrus; Cun, Cuneus; AG, Angular Gyrus.*

### Correlation Between Local Metrics of rs-fMRI and Clinical Variables

**FIGURE 4:**
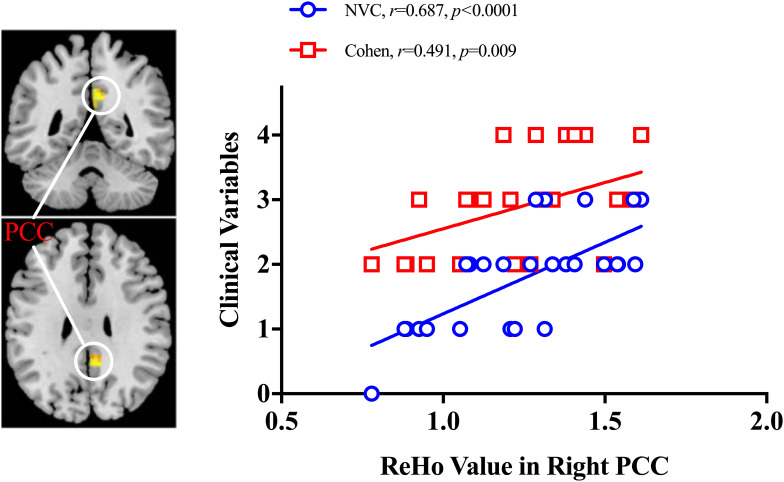
Correlation analyses between ReHo value and clinical variables. The positive correlations were observed between ReHo value in right posterior cingulate cortex and NVC degree and Cohen spasm scale in HFS patients. PCC, posterior cingulate cortex; NVC, neurovascular compression.

**FIGURE 5:**
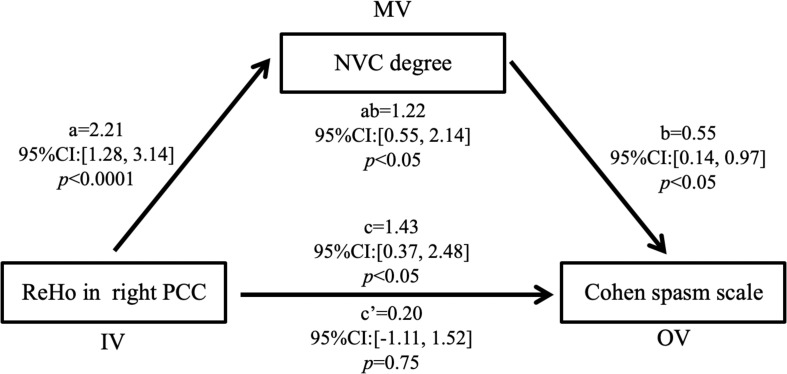
NVC degree completely mediated the relationship between ReHo in the right PCC and Cohen spasm scale. The results suggested that ReHo in the right PCC may have an indirect effect on the Cohen spasm scale predominantly through NVC. Unstandardized regression coefficients were shown in the figure. IV, independent variable; MV, mediator variable; OV, outcome variable; PCC, posterior cingulate cortex; NVC, neurovascular compression.

As shown in Figure 4, ReHo in the right PCC was positively correlated with NVC degree (*r* = 0.687, *p* < 0.0001) and Cohen spasm scale (*r* = 0.491, *p* = 0.009) in the HFS patients. No other significant correlation was found between fALFF/ReHo/DC and Cohen/NVC scores in the remaining ROI masks. Furthermore, in the mediation analysis, if ReHo in the right PCC was regarded as the mediator variable, the path coefficients a and c’ were both significant (*p* < 0.05), b [95%CI: (−1.11, 1.520)] and a × b were non-significant [95%CI: (−0.18, 0.28)], so neurovascular compression had a direct effect on the Cohen spasm scale not being mediated by ReHo in the right PCC. Correspondingly, if NVC degree was regarded as the mediator variable, it would have a completely mediating effect on the relationship between ReHo in the right PCC and Cohen spasm scale with path coefficients a, b, and a × b being all significant (*p* < 0.05), and c’ being non-significant [95%CI: (−1.11, 1.52)] (see Figure 5). These results suggested that ReHo in the right PCC tend to have an indirect effect on spasm ratings predominantly through the alteration of neurovascular compression.

## Discussion

In this study, we applied fALFF, ReHo, and DC to investigate functional abnormalities of local brain in HFS patients, and further explored the correlations between these abnormalities with clinical variables. Compared with healthy controls, HFS patients exhibited increased fALFF in the left precuneus and right PCC. The brain regions showing increased ReHo were mainly located in the bilateral PCC and bilateral precuneus, together with decreased ReHo in the right MOG, right SOG, right cuneus, and right AG. Moreover, ReHo in the right PCC was positively correlated with the NVC degree and Cohen spasm scale in HFS patients, respectively. Mediation analysis revealed ReHo in the right PCC indirectly affected spasm ratings predominantly through the alteration of NVC. To the best of our knowledge, this is the first study applying fALFF, ReHo, and DC simultaneously to detect the regional brain functional alterations in HFS patients, and the findings indicate that PCC dysfunctions may be a factor of microvascular decompression failure and cause spasm recurrence by affecting neurovascular compression, which provides a new insight for understanding the neuropathological mechanisms of HFS.

As a most direct measure of voxel-wise local centrality, DC reflects node characteristic of cortical network architecture, which could be used to reveal the complexity of brain function connectivity networks as a whole. Previous studies have observed that certain brain regions acting as hubs were prone to disconnection and dysfunction using DC analysis (Buckner et al., 2009; Zuo et al., 2012). However, in our study, with correlation coefficient threshold r*_*ij*_* > 0.25 (Buckner et al., 2009), DC could not detect the difference between HFS patients and healthy controls. Generally, DC showed lower performance (e.g., test-retest reliability) in comparison with other local metrics, such as ALFF (Wang et al., 2017). Furthermore, DC is inclined to be affected by various experimental and analytical strategies including computational space, head motion, and global signal regression (Zuo and Xing, 2014). Global signal regression even had a crucial influence on spatial pattern of DC (Liao et al., 2013; Murphy and Fox, 2016). Therefore, it is inferred that poor stability and vulnerability to interference affected the detection performance of DC on local brain abnormality in HFS patients.

As a normalized indicator of ALFF, fALFF measures the intensity of spontaneous neuronal activity corresponding to a single voxel, selectively suppressing artifacts from perivascular, periventricular, and periaqueductal regions (He et al., 2007; Zuo et al., 2010). ReHo characterizes the regional neural activity synchronization in the nearest neighbors of a voxel, which reflects the local properties in a larger region compared to fALFF. As shown in this study, ReHo detected more abnormal brain regions in HFS patients than fALFF, which indicated that these voxel-wised metrics complement each other to offer a comprehensive delineation of regional brain abnormalities (An et al., 2013; Lv et al., 2019). Specifically, precuneus and PCC not only exhibited increased fALFF, but also increased ReHo in HFS patients compared to healthy controls, which were partly consistent with the previous findings (Tu et al., 2015). The increased fALFF and ReHo indicated an enhanced spontaneous neuronal activity and local synchronization in these regions. As is well known, PCC/precuneus is part of the brain regions that support executive control of movement initiation, which has been proved in other movement disorders (Jiang et al., 2019; Spay et al., 2019). A previous rs-fMRI study on humans and macaque monkeys also indicated that the anterior precuneus is part of the sensorimotor processing zone as it was functionally connected with the superior parietal cortex, paracentral lobule, and motor cortex (Margulies et al., 2009). Precuneus contribute to sensorimotor integration by combining multiple sensory inputs such as somatosensory and visual input, and involve in forming movement intention or early movement plans (Andersen and Buneo, 2002; Asher et al., 2015; Whitlock, 2017). Moreover, as part of the integrative center, PCC also participates in the motor and attention processes (Pearson et al., 2011; Reynolds et al., 2017). Therefore, the elevated fALFF and ReHo may consistently reflect compensatory neural adaptation of the PCC/precuneus for the movement control in HFS patients. Nevertheless, the increased neuronal activity of local brain in HFS patients seems to be not enough to prevent facial spasm.

The positive correlation of ReHo in right PCC to neurovascular compression degree and spasm severity were observed in our study. These regional neural activity synchronizations in right PCC may provide an alternative index to monitor the neurovascular compression degree and spasm severity of HFS patients. In addition, previous study has reported a positive correlation of Cohen spasm scale with NVC degree (Bao et al., 2015). In consideration of these associations, it was worth to determine which one played a dominant role on spasm severity. By applying mediation analysis, we found the relationship between ReHo in the right PCC and Cohen spasm scale was completely mediated by NVC degree. This implied that abnormal ReHo in the right PCC tend to regulate the change in the neurovascular compression, and further affect facial spasm ratings, which revealed the ReHo in the right PCC might be a cause of HFS disease and a factor of the failure in microvascular decompression and spasm recurrence.

Our study also demonstrated decreased ReHo in MOG, SOG, cuneus, and AG in HFS patients. Most of these regions are part of the occipital lobe, belonging to the visual information processing center of the mammalian brain (Machielsen et al., 2000; Van Essen, 2003; Thiebaut de Schotten et al., 2014). Furthermore, considering that facial spasm usually originates from the orbicularis oculi muscle, and frequent eyelid closure of HFS patients may cause visual disability (Wang and Jankovic, 1998; Tan et al., 2004), it is inferred that these decreased ReHo in occipital lobe may be linked to visual processing deficit.

A few limitations should be noted in this study. First, longitudinal investigations are needed to further verify the indirect effect of ReHo in the right PCC as a probable cause of HFS disease on spasm ratings. Second, the medication might affect the local brain function. However, drug effects are inevitable and difficult to be entirely removed due to various medication regimens among each HFS patient. Third, future researches will explore the test-retest reliability and reproducibility of these results across multiple cohorts and apply other methods to find more reliable and reproducible results.

## Conclusion

In summary, this study revealed the regional functional alterations of the brain through fALFF, ReHo, and DC analyses in HFS patients. The correlations were also detected between ReHo in the right PCC and Cohen spasm scale and NVC degree, indicating a potential link between facial nerve and cerebral cortex. Furthermore, the relationship between ReHo in the right PCC and Cohen spasm scale was completely mediated by NVC degree. These findings may imply local cerebral functional disruptions of HFS patients and improve the understanding of the pathophysiological mechanisms of the disease.

## Data Availability Statement

The raw data supporting the conclusion of this article will be made available by the authors, without undue reservation.

## Ethics Statement

The studies involving human participants were reviewed and approved by Ethics Committee at the First Affiliated Hospital of Xi’an Jiaotong University. The patients/participants provided their written informed consent to participate in this study.

## Author Contributions

YW and MZ proposed and supervised the project. YW and HX collected the data. F-FL and HX processed and analyzed the data and wrote the manuscript. All authors revised the manuscript and approved the final version.

## Conflict of Interest

The authors declare that the research was conducted in the absence of any commercial or financial relationships that could be construed as a potential conflict of interest.
